# The impact of adverse childhood experiences on depression in old age: evidence from China

**DOI:** 10.3389/fpsyt.2025.1626389

**Published:** 2025-10-16

**Authors:** Ziqiong Liu, Enlin Cao, Hua Wei

**Affiliations:** ^1^ School of Economics and Management, Anhui University of Chinese Medicine, Hefei, Anhui, China; ^2^ Key Laboratory of Data Science and Innovative Development of Chinese Medicine in Anhui Province, Philosophy and Social Sciences, Hefei, Anhui, China; ^3^ School of Finance and Public Management, Anhui University of Finance & Economics, Bengbu, Anhui, China

**Keywords:** adverse childhood experiences (ACEs), older adults, offspring support, depression, old-age pension

## Abstract

**Objective:**

This study explores the links between negative childhood experiences and depression in older adults, focusing on how pensions and offspring support influence the relationship between adverse childhood experiences (ACEs) and the mental health outcomes of the elderly.

**Methods:**

Data were obtained from the 2016, 2018, and 2020 China Longitudinal Aging Social Survey (CLASS). We used the ordinary least squares (OLS) method and moderation tests to analyze depression health outcomes.

**Results:**

Older adults with ACEs had worse mental health outcomes than those without ACEs. The ACEs of medical deficiency, parental absence, and hunger during childhood manifested as higher depression scores in old age. The heterogeneity regression results show that medical deficiency and hunger experiences have a more significant impact on the depression of elderly individuals in urban areas. In contrast, early experiences of parental absence have a greater effect on the depression of elderly individuals in rural areas. Regression results for moderating effects indicate that children’s support can effectively alleviate the impact of adverse childhood experiences on the depression status of the elderly; however, pensions and the number of friends do not play a positive moderating role.

**Conclusion:**

ACEs, pension, and offspring support were independently associated with older adults’ mental health problems, and the combination of ACEs and low offspring support was the most significant predictor of adverse health outcomes in old age, controlling for adult sociodemographic indicators. Enhancing supportive relationships between children and older adults may buffer the negative effects of early adversities on older adult well-being.

## 1 Introduction

Globally, population aging is an inevitable demographic trend that presents major challenges to public health systems, especially regarding mental health in older adults. Depression is a common and debilitating condition among seniors (1, 2). According to the World Health Organization, over 10% of older adults worldwide experience depression, significantly contributing to the global disease burden (3). This issue is even more severe in low- and middle-income countries experiencing rapid demographic shifts.

China exemplifies this challenge. As the world’s most populous nation, it is also aging at an unprecedented pace. Data from the National Bureau of Statistics of China (2024) reveals that 21.1% of the population (296.97 million) is aged 60 and above (4). Alarmingly, the prevalence of mental health issues, especially depression, among Chinese older adults is remarkably high. A large-scale meta-analysis estimated that the pooled prevalence of depressive symptoms in adults aged 60 and above in China is 37.9% (5), far surpassing the global average. Significant urban-rural disparities exist, with rural elders often experiencing worse mental health outcomes (6). This stark reality highlights the urgent need to identify risk factors and protective mechanisms for late-life depression in the Chinese context.

To effectively address this issue, a life course perspective offers a valuable theoretical view. This framework indicates that health in old age is not just a current state but is shaped by a lifetime of experiences, exposures, and accumulated advantages or disadvantages (7, 8). Early-life hardships, in particular, can have enduring effects, influencing health trajectories through biological embedding and socioeconomic pathways (9, 10). Research consistently shows that adverse childhood experiences (ACEs), such as abuse, neglect, and household dysfunction, are important predictors of poor physical and mental health outcomes throughout life, including depression in old age (11, 12).

However, the impact of ACEs is not necessarily deterministic. The life course model also highlights the potential for resilience and the moderating influence of resources available later in life (13). Factors such as financial security (e.g., pension income) and social support can buffer the negative effects of early-life adversities (14, 15). While the link between ACEs and poor health is well-established in Western contexts, less is known about this relationship and its potential moderators within China’s unique socio-cultural and economic environment, especially among its large aging population (16, 17).

Therefore, this study aims to investigate the connection between adverse childhood experiences and depression in later life using a large, nationally representative sample of Chinese older adults. Based on life course theory, it also examines whether later-life resources—specifically pension income and support from adult children—can reduce the long-term psychological impact of ACEs. The findings will provide crucial evidence for developing targeted interventions to enhance mental well-being among Chinese older adults who experienced early-life hardships.

## 2 Literature review

### 2.1 Multifaceted influencing factors of late-life depression

Depression in older adults arises from various interconnected biological, psychological, social, and environmental factors. These factors together worsen mental health challenges for seniors and tend to build up over a lifetime.

Biological and health-related factors greatly influence geriatric depression. Older adults often encounter chronic illnesses—such as cardiovascular disease, diabetes, and functional limitations—that directly contribute to depressive symptoms (18, 19). Moreover, cognitive impairment is widely recognized as a factor related to late-life depression (20–26). Health behaviors like smoking and substance abuse also raise the risk of depression (27–29).

Socio-demographic and economic factors are strongly associated with depression rates. Studies consistently indicate that female gender, older age, lower education levels, unemployment, and low income increase vulnerability (20–22, 24, 30–35). Marital status — such as being single or divorced — and living alone are also significant psychosocial risk factors (30, 35). Additionally, lacking health insurance further exacerbates these disparities (35).

Psychosocial and environmental factors are equally vital. Reduced social roles after retirement, less social interaction, loneliness, and bereavement significantly contribute to depression (22, 23, 37–40). Family support, living conditions, and socioeconomic status further affect depression risk (21, 26, 41–43). Large-scale events like economic shifts and public health crises also have notable impacts (44, 45).

Beyond immediate factors, a life-course perspective emphasizes that early-life experiences—such as childhood trauma (46), low self-esteem, and adverse events—can set patterns of vulnerability that lead to depression in later years. This approach combines distant and recent factors, providing a more complete understanding of geriatric depression.

### 2.2 The long shadow: childhood experiences and depression in old age

Among the many factors that influence depression, childhood experiences are an important and often overlooked cause of depression in older adults. Childhood is a key stage of psychological, emotional, and social development, and the mental health of the elderly is inevitably shaped by early childhood experiences.

Experiencing more ACEs raises the risk of developing major depression and depressive symptoms in adulthood (47).Research consistently shows that adverse childhood experiences (ACEs), such as abuse, neglect, and household dysfunction, are strong predictors of poor physical and mental health outcomes throughout life, including depression in old age (11, 12).

Specifically, experiences of childhood hunger can harm self-rated health, functional health, and cognitive health in the elderly (48). Moreover, a lack of emotional support during early life can lead to depression later in life. Previous research has indicated that childhood foster care experience and emotional neglect are strongly linked to depression in later years (49, 50).

### 2.3 Mechanisms linking ACEs to late-life depression

While the academic community generally agrees that ACEs increase the likelihood of depression in older adults, scholars hold diverse views on how this occurs.

Biological mechanisms offer one explanation. Eleonora et al. suggest that inflammation might be a psychobiological pathway connecting ACEs and depression (51). Emerging evidence shows that biological aging could be an important mediator linking ACEs and depression (52, 53). Building on this idea, Campbell et al. proposed that the physiological stress response caused by ACEs may negatively impact the nervous, neuroendocrine, and immune systems, potentially leading to physical and mental health issues later in life, such as toxic stress and allostatic load (54).

Psychosocial pathways provide complementary explanations. From a sociological perspective, evidence shows that life satisfaction partially mediates the relationship between childhood socioeconomic status (SES) and depressive symptoms in middle and old age. Improving life satisfaction can serve as an intervention to reduce future depressive symptoms (55). Focusing on the period from early life to old age, socioeconomic status in adulthood can partially mediate this relationship, and achieving higher adult SES can help lessen the negative effects of ACEs on depression later in life through exposure interactions (56).

The mechanisms involving family support are less studied. There is no clear article on whether family support or children’s emotional communication mediates the relationship between ACEs and depression in the elderly. A related article suggested that when family emotional support is high, it provides a stronger protective effect against the impact of loneliness on depression (57).

### 2.4 Theoretical framework, research gaps, and hypotheses

Grounded in life course theory (7, 58) and cumulative disadvantage theory (59), this study constructs an integrative framework to examine how adverse childhood experiences influence late-life depression in China and how later-life resources might buffer this relationship.

Existing studies have extensively documented the impact of childhood adversities on depression later in life, providing a valuable foundation for this research. However, some aspects remain less thoroughly explored within specific populations. While the link between ACEs and poorer health outcomes is well-documented in Western societies (60), its manifestation and potential moderating mechanisms within China’s unique socioeconomic and cultural contexts—especially among older adults—require further empirical investigation (61). Additionally, the role of children’s support in reducing late-life depression, particularly as a buffer against the long-term effects of ACEs, has received comparatively limited scholarly attention (62).

The life course model emphasizes the potential for resilience and the moderating role of resources available in later life (13). Factors such as financial security and social support may buffer the negative effects of early-life adversities.

Therefore, this study aims to further investigate the impact of childhood adversities on depression in older adults and, more importantly, to determine whether and how resources in later life can lessen this relationship. Based on the theoretical framework and identified research gaps, we propose the following hypotheses.

H1: Adverse childhood experiences (ACEs) are linked to higher levels of depressive symptoms among older Chinese adults.

H2: Pension income will weaken the positive link between ACEs and depressive symptoms.

H3: Offspring support will weaken the positive link between ACEs and depressive symptoms.

H4: The links between ACEs and depression will differ between urban and rural residents.

**Figure 1:**
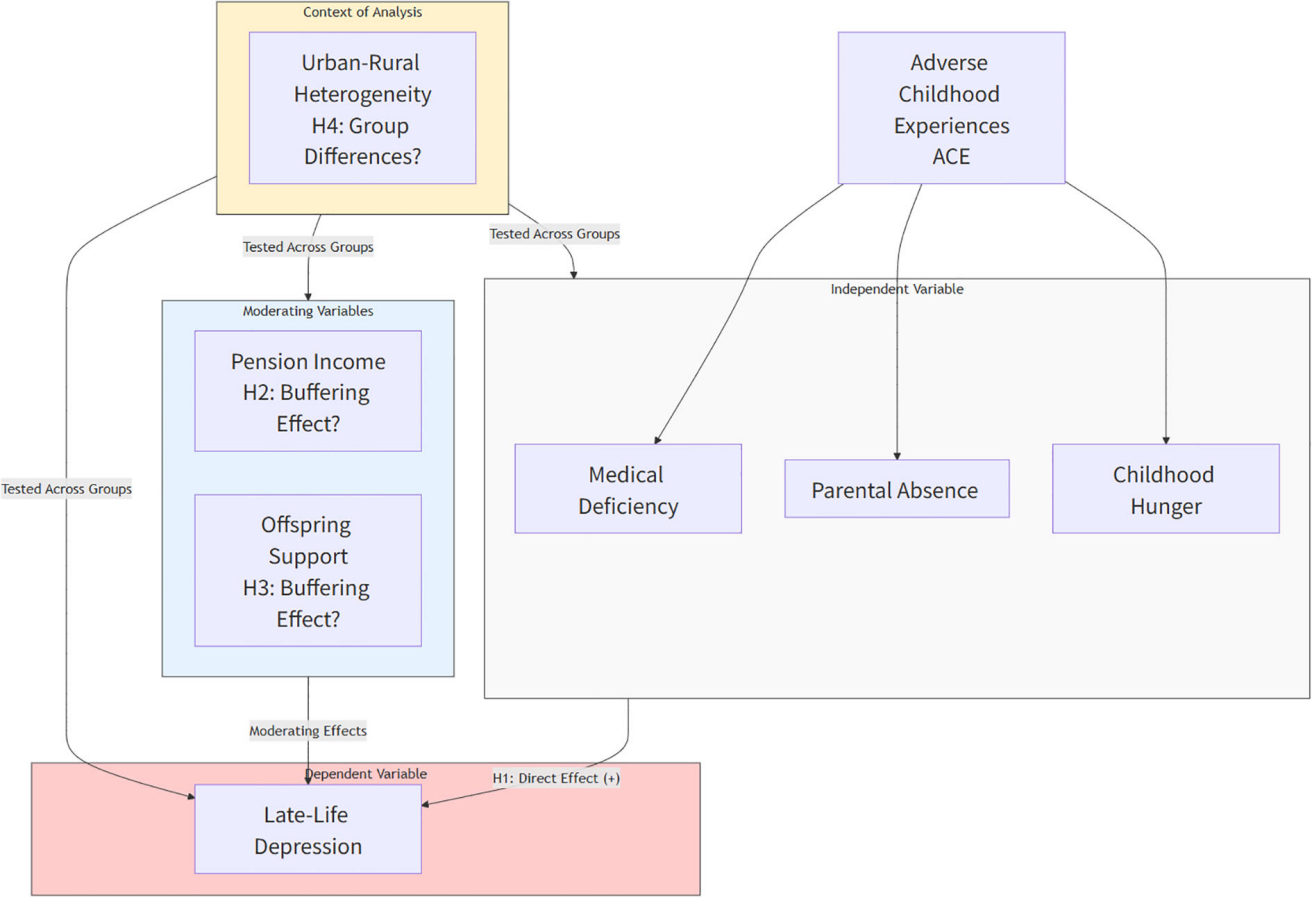
Theoretical framework diagram of the impact of adverse childhood experiences on depression in the elderly.

Based on the outlined theoretical foundations and hypotheses, we developed an integrated conceptual framework (**Figure 1**) to visually illustrate the proposed relationships among adverse childhood experiences, resources in later life, and depressive symptoms in older adults, specifically within China’s urban and rural contexts.

## 3 Materials and methods

### 3.1 Data source

The 2016, 2018, and 2020 three-year follow-up survey data from the China Longitudinal Aging Social Survey (CLASS) were used in this study. A nationwide longitudinal sociological survey project is managed and carried out by China’s Renmin University. CLASS’s main goal is to gain a thorough understanding of the aged population’s situation. Twenty-eight provinces, including municipalities and autonomous regions, were surveyed. All procedures involving human participants were performed according to the ethical standards of the institutional and/or national research committee and in line with the 1964 Declaration of Helsinki and its later revisions or comparable ethical standards. The survey was also conducted in Articles 38, 39, and 40 of the Constitution of the People’s Republic of China and within the legal framework governed by Chapter I, Article 9 of the Statistics Law of the People’s Republic of China. Therefore, this study was not approved by an ethics committee. Verbal informed consent was obtained from all individual participants involved in the study. The survey design adhered to Articles 38, 39, and 40 of the Constitution of the People’s Republic of China and the legal framework outlined by Chapter I, Article 9 of the Statistics Law of the People’s Republic of China. Verbal informed consent was deemed acceptable and did not require review by an ethics committee. Additionally, the interviewer documented detailed information regarding the process of obtaining consent, including whether participants agreed to participate, the time of consent, reasons for refusal, etc. These consent details were stored by the Institute of Gerontology and the National Survey Research Center at Renmin University of China. The survey included numerous elements related to the individual, familial, and social characteristics of the elderly, using a hierarchical multistage random sampling approach. This includes analyzing the ACEs of older adults and their mental health. As a result, the survey provides a broad national sample, enabling a comprehensive analysis of seniors’ ACEs and depression.

The sample selection followed these criteria: (1) Only individuals with complete follow-up data for all three years and no missing key variables were included; (2) Observations with missing key variables, logical inconsistencies, or extreme outliers were excluded; (3) The final balanced panel dataset consisted of 9,675, 9,895, and 9,881 observations for the years 2016, 2018, and 2020, respectively, totaling 29,451 cases. This nationally representative sample effectively supports inferences about the relationship between adverse childhood experiences and depression in later life.

### 3.2 Variables

#### 3.2.1 Dependent variable: depressive symptoms

Depressive symptoms were measured with an 11-item short form of the Center for Epidemiologic Studies Depression Scale (CES-D), which is included in the CLASS questionnaire. This validated scale is commonly used in aging research to assess how often depressive feelings occur over the past week. The total score was computed so that higher scores reflect more severe depressive symptoms. A detailed overview of all 11 items, response choices, and the scoring method—including how positive items are reverse-coded—is available in Appendix A.

#### 3.2.2 Core independent variable: adverse childhood experiences

The main explanatory variable was older adults’ exposure to adverse childhood experiences (ACEs). Guided by the life-course perspective, which suggests that early-life adversity can have long-term health effects, we created a composite measure of ACEs based on three dimensions common in the historical context of the study population: (1) medical deficiency, (2) parental absence, and (3) childhood hunger (experiencing frequent food scarcity). These dimensions represent key aspects of material and psychosocial deprivation that are believed to influence mental health trajectories in later life.

#### 3.2.3 Control variables

The study includes several control variables, such as age, gender, marital status, household registration type (rural or urban), presence of chronic diseases, internet access, recent exposure to major accidents, housing type, the number of family members living in the household, availability of free health check-ups in the community, and basic community infrastructure.

#### 3.2.4 Mediating variables

To explore potential pathways connecting adverse childhood experiences (ACEs) to depression in later life, we included two mediating variables: pension income and child support. Pension income was measured as a binary variable indicating whether the respondent received a steady monthly pension from an employer or government agency, representing a stable post-retirement financial situation. Child support was assessed based on the frequency and value of monetary or in-kind transfers from children over the past 12 months, reflecting the level of family support in old age. The reason for examining these mediators is that ACEs may affect mental health through their long-term impact on socioeconomic status and the quality of social relationships in adulthood.

For specific definitions and measurement methods of these variables, please see **Table 1**.

**Table 1:** Variable descriptive statistics.

Variable	Variable definition	Mean	Standard deviation
Explained variable			
Depression	Calculated according to the scale, the higher the score, the greater the degree of depression	30.293	14.020
Loneliness	Yes=1,no=0	0.452	0.498
Explanatory variable			
medical deficiency	During childhood, access to healthcare services was unavailable. Yes=1,no=0	0.791	0.407
parental absence	At the age of ten, at least one parent had passed away. Yes=1,no=0	0.100	0.299
hunger	Experiencing frequent hunger during childhood. Yes=1,no=0	0.640	0.480
Individual characteristics			
Age	Chronological age of the older adults	70.802	7.099
Gender	Males = 1, females = 0	0.506	0.500
Marriage	Married = 1, unmarried = 0	0.728	0.445
Registration	Rural = 1, urban = 0	0.509	0.500
Chronic diseases	Yes = 1, no = 0	0.654	0.476
Internetuse	Yes = 1, no = 0	0.485	0.500
Significant accidents	Yes = 1, no = 0	0.181	0.385
Type of housing	Building = 1, Other = 0	0.557	0.497
Number of permanent household residents	The number of individuals residing permanently within a household.	2.676	1.281
Free medical check-up	Yes = 1, no = 0	0.282	0.450
Community facilities	Yes = 1, no = 0	0.794	0.405
Potential moderating variables			
Pension	Yes = 1, no = 0	0.352	0.478
Offspring support	The quantity of support received by the elderly from their children.	2.089	1.363

### 3.3 Statistical methods

All statistical analyses were conducted using Stata version 17.0. Given the longitudinal nature of the data, a panel fixed-effects regression model was employed to account for time-invariant, unobservable individual differences.

The mental health index for older individuals, developed in this study, measures depression and is expressed as a continuous variable. Therefore, the initial estimation in the benchmark regression analysis is performed using the ordinary least squares (OLS) method. The fundamental formula for OLS is as follows:


(1) $$Depression_{i,t}=\alpha_{1}+\alpha_{2}ACEs_{i,t}+\beta_{t}X_{i,t}+\epsilon_{i,t}$$

$Depression_{i,t}$ in Equation 1 indicates depression in older individuals. $ACEs_{i,t}$ is adverse childhood experiences of the older people surveyed and is the explanatory variable that this article focuses on. $X_{i,t}$ represents all other control variables, $\epsilon_{i,t}$ is the random error term, $\alpha_{1}$ is the constant term, $\alpha_{2}$ represents the coefficient of the number incorporated, and $\beta_{t}$ is the parameter to be estimated for the other control variables. Let i denote elderly individuals, and t represent different years.

There may be selection bias between ACEs and depression in older adults. The ACEs of older people depend on their circumstances, not randomly, which causes a problem of selection bias. Additionally, individual characteristics of older adults can be linked to both their ACEs and depression, making ordinary least squares estimation prone to bias. To address this potential selectivity bias, this chapter uses propensity score matching (PSM) to measure the net effect of ACEs on depression in older adults. The model divides the sample into an experimental group and a control group and matches them using a propensity score for phase-based analysis to minimize the influence of other factors. The specific model is as follows:


(2) $$Depression_{i,t}=Depression_{0i,t}+\left(Depression_{1i,t}-Depression_{0i,t}\right)D_{i,t}$$

(3) $$ATT=E\left(Depression_{1i,t}-Depression_{0i,t}|D_{i,t}=1\right)$$

In Equation 2, $D_{i,t}$ is the treatment variable. When $D_{i,t}$ equals 1, it indicates that individual i belongs to the experimental group and participates in the intervention project in t year. Conversely, when $D_{i,t}$ equals 0, it indicates that individual i belongs to the control group and does not participate in the intervention project in t year. In this study, the core independent variables are divided into two groups. The experimental group comprises older adults with ACEs, while the control group consists of older individuals without ACEs. Equation 3 denotes the average treatment effect observed in the experimental group. It quantifies the net effect of ACEs on the depression of older adults.

The life course model posits that adverse childhood experiences may influence health in old age by affecting social experiences during adulthood, such as education and economic status (i.e., adult socioeconomic conditions affect health investments). To examine this hypothesis, this study builds upon the baseline regression to conduct a moderating effect analysis:


(4) $$Depression_{i,t}=\alpha_{1}+\alpha_{2}ACEs_{i,t}+\alpha_{3}Z_{i,t}+\alpha_{4}ACEs_{i,t}\times Z_{i,t}+\beta_{t}X_{i,t}+\epsilon_{i,t}$$

In Equation 4, $Z_{i,t}$ represents the moderating variable and $\alpha_{4}ACEs_{i,t}\times Z_{i,t}$ denotes the interaction term.

## 4 Results

### 4.1 Benchmark regression results

**Table 2** displays the results of a benchmark regression analysis examining the link between ACEs and depression among older adults. The analysis carefully includes a set of control variables to examine the relationship. Medical deficiency, parental absence, and childhood hunger significantly increase depressive symptoms in older adults. In all models, the coefficients for numerical integration consistently show positive values, indicating a statistically significant improvement in mood at a 1% significance level. These regression results offer strong evidence supporting the positive relationship between various ACEs and the emotional well-being of older adults.

**Table 2:** Relationship between ACEs and depression of older adults(OLS).

Model	(1)	(2)	(3)	(4)
Medical deficiency	2.527***			2.002***
	(0.198)			(0.198)
Parental absence		1.921***		1.741***
		(0.248)		(0.246)
Hunger			2.873***	2.548***
			(0.171)	(0.173)
Age	0.137***	0.140***	0.131***	0.123***
	(0.012)	(0.012)	(0.012)	(0.012)
Gender	-0.727***	-0.728***	-0.772***	-0.785***
	(0.157)	(0.158)	(0.157)	(0.157)
Marriage	-1.753***	-1.755***	-1.757***	-1.701***
	(0.186)	(0.186)	(0.185)	(0.185)
Registration	0.223	0.226	0.097	0.098
	(0.186)	(0.187)	(0.186)	(0.185)
Chronic diseases	0.886***	0.950***	0.903***	0.855***
	(0.171)	(0.171)	(0.170)	(0.170)
Internetuse	-3.936***	-4.139***	-3.993***	-3.913***
	(0.262)	(0.263)	(0.261)	(0.261)
Significant accidents	3.313***	3.359***	3.138***	2.996***
	(0.211)	(0.210)	(0.211)	(0.211)
Type of housing	-1.344***	-1.444***	-1.330***	-1.258***
	(0.184)	(0.184)	(0.184)	(0.184)
Number of permanent household residents	-0.268***	-0.243***	-0.294***	-0.316***
	(0.062)	(0.062)	(0.062)	(0.062)
Free medical check-up	-0.273	-0.181	-0.386**	-0.367**
	(0.186)	(0.187)	(0.186)	(0.185)
Community facilities	-2.147***	-2.109***	-2.200***	-2.191***
	(0.211)	(0.211)	(0.211)	(0.211)
Year	YES	YES	YES	YES
Region	YES	YES	YES	YES
Constant	20.819***	21.939***	21.539***	20.949***
	(1.107)	(1.109)	(1.102)	(1.100)
R-squared	0.141	0.138	0.145	0.149
N	29,451	29,451	29,451	29,451

***p<0.01, **p<0.05, *p<0.1; Robust standard errors in parentheses.

### 4.2 Robustness test

To ensure the reliability of our benchmark regression results, we used both the substitution variable method. Given the close link between loneliness and depression among the elderly, we performed a robustness check by replacing the dependent variable with loneliness. The regression results in **Table 3** show that medical deficiency, parental absence, and childhood hunger significantly increase loneliness among older adults. Including these three adverse childhood experiences (ACEs) in the model consistently reveals that each one substantially raises feelings of loneliness in later life.

**Table 3:** Robustness test - replacement as dependent variable (logit).

Model	(1)	(2)	(3)	(4)
Variables	Loneliness			
Medical deficiency	0.216***			0.179***
	(0.032)			(0.032)
Parental absence		0.116***		0.103**
		(0.041)		(0.041)
Hunger			0.214***	0.187***
			(0.027)	(0.027)
Age	0.007***	0.007***	0.007***	0.006***
	(0.002)	(0.002)	(0.002)	(0.002)
Gender	0.013	0.013	0.010	0.009
	(0.025)	(0.025)	(0.025)	(0.025)
Marriage	-0.577***	-0.577***	-0.578***	-0.574***
	(0.030)	(0.029)	(0.030)	(0.030)
Registration	0.027	0.028	0.018	0.018
	(0.030)	(0.030)	(0.030)	(0.030)
Chronic diseases	-0.028	-0.023	-0.026	-0.030
	(0.027)	(0.027)	(0.027)	(0.027)
Internetuse	-0.323***	-0.338***	-0.328***	-0.320***
	(0.040)	(0.040)	(0.040)	(0.040)
Significant accidents	0.313***	0.319***	0.301***	0.291***
	(0.032)	(0.032)	(0.032)	(0.032)
Type of housing	-0.139***	-0.147***	-0.139***	-0.133***
	(0.030)	(0.030)	(0.030)	(0.030)
Number of permanent household residents	-0.080***	-0.078***	-0.082***	-0.084***
	(0.010)	(0.010)	(0.010)	(0.010)
Free medical check-up	0.046	0.053*	0.038	0.039
	(0.029)	(0.029)	(0.029)	(0.029)
Community facilities	-0.145***	-0.142***	-0.148***	-0.149***
	(0.034)	(0.034)	(0.034)	(0.034)
Year	YES	YES	YES	YES
Region	YES	YES	YES	YES
Constant	-0.038	0.055	0.025	-0.033
	(0.176)	(0.176)	(0.176)	(0.176)
Observations	29,451	29,451	29,451	29,451

***p<0.01, **p<0.05, *p<0.1; Robust standard errors in parentheses.

### 4.3 Counterfactual test based on propensity score matching

This section uses propensity score matching (PSM) to address self-selection bias and estimate the true effect of ACEs on the mental health of older adults. Specifically, the chapter examines ACEs among the elderly. The control group includes older individuals with no adverse childhood experiences (ACEs), while the treatment group consists of those who have experienced ACEs. Three common PSM methods are employed: nearest neighbor matching, radius matching, and kernel matching. The sample balance test based on nearest neighbor matching shows that, compared to before matching, the standard deviation (% bias) of all variables after matching is under 10%, and the differences in sample features are greatly reduced, indicating that the matching effectively balances the data.

**Table 4** shows the results of propensity score matching examined to assess the impact of Adverse Childhood Experiences (ACEs) on depression levels in older adults. The ATT values of 1.7855, 1.7020, and 2.5647 for medical absence, parental absence, and starvation, respectively, estimated through nearest-neighbor matching, were all significant at the 1% level. This indicates that these ACEs notably increase depressive symptoms among older adults. The study was further confirmed using radius matching with kernel matching, and the results aligned with those from nearest neighbor matching. The findings support the baseline regression coefficients, reaffirming the strong influence of ACEs on increasing depression levels in older adults.

**Table 4:** Retest of the propensity score matching method based on counterfactual inference.

Variable name	Matching method	ATT	Standard deviation	T statistics
Medical deficiency	Nearest Neighbor Matching(1:4)	1.7855	0.2932	6.09***
	Radius Matching(0.05)	2.0563	0.2519	8.16***
	Kernel Matching(Default)	2.0875	0.2498	8.36***
Parental absence	Nearest Neighbor Matching(1:4)	1.7020	0.2988	5.7***
	Radius Matching(0.05)	2.2155	0.2648	8.37***
	Kernel Matching(Default)	2.2100	0.2653	8.33***
Hunger	Nearest Neighbor Matching(1:4)	2.5647	0.2334	10.99***
	Radius Matching(0.05)	2.7268	0.2107	12.94***
	Kernel Matching(Default)	2.7339	0.2112	12.94***

***p<0.01, **p<0.05, *p<0.1.

Based on the benchmark regression analysis, the study gradually added control variables and conducted various robustness tests, including propensity score matching, to reduce self-selection bias. The results consistently show that adverse childhood experiences (ACEs) significantly increase depression among older adults. Therefore, it is reasonable to cautiously conclude that ACEs substantially contribute to the higher likelihood of depression in older adults.

### 4.4 Heterogeneity analysis

**Table 5** shows the regression results of the heterogeneity analysis. The study indicates that adverse childhood experiences (ACEs) early in life increase depressive scores in later years among both rural and urban elderly populations. However, the effects of specific ACEs differ between these regions. Specifically, inadequate childhood medical care and experiences of hunger have a stronger impact on depression among the urban elderly, while early parental absence has a greater influence on depressive symptoms in the rural elderly.

**Table 5:** Heterogeneity analysis.

Model	(1)	(2)	(3)	(4)	(5)	(6)
	Rural	Urban	Rural	Urban	Rural	Urban
Medical deficiency	1.064***	3.635***				
	(0.291)	(0.271)				
Parental absence			2.325***	1.219***		
			(0.320)	(0.383)		
Hunger					1.338***	4.060***
					(0.242)	(0.240)
Age	0.097***	0.177***	0.094***	0.188***	0.096***	0.165***
	(0.017)	(0.017)	(0.017)	(0.017)	(0.017)	(0.017)
Gender	-0.664***	-0.796***	-0.674***	-0.785***	-0.681***	-0.849***
	(0.214)	(0.230)	(0.214)	(0.231)	(0.214)	(0.229)
Marriage	-1.993***	-1.533***	-1.968***	-1.570***	-1.998***	-1.516***
	(0.250)	(0.274)	(0.250)	(0.276)	(0.250)	(0.273)
Chronic diseases	0.331	1.458***	0.358	1.565***	0.337	1.490***
	(0.236)	(0.247)	(0.236)	(0.249)	(0.236)	(0.246)
Internetuse	-4.558***	-3.906***	-4.654***	-4.216***	-4.558***	-4.051***
	(0.450)	(0.330)	(0.450)	(0.332)	(0.451)	(0.328)
Significant accidents	3.262***	3.276***	3.239***	3.428***	3.174***	3.061***
	(0.287)	(0.310)	(0.287)	(0.309)	(0.288)	(0.309)
Type of housing	-1.035***	-2.012***	-1.061***	-2.176***	-0.989***	-2.050***
	(0.249)	(0.300)	(0.248)	(0.302)	(0.248)	(0.301)
Number of permanent household residents	-0.223***	-0.335***	-0.213**	-0.296***	-0.231***	-0.395***
	(0.086)	(0.091)	(0.085)	(0.091)	(0.086)	(0.091)
Free medical check-up	-1.208***	0.445*	-1.104***	0.548**	-1.213***	0.133
	(0.262)	(0.264)	(0.263)	(0.267)	(0.262)	(0.266)
Community facilities	-2.308***	-2.253***	-2.288***	-2.136***	-2.333***	-2.362***
	(0.261)	(0.379)	(0.261)	(0.378)	(0.261)	(0.375)
Year	YES	YES	YES	YES	YES	YES
Region	YES	YES	YES	YES	YES	YES
Constant	25.782***	17.296***	26.389***	18.530***	25.786***	18.930***
	(1.561)	(1.550)	(1.554)	(1.564)	(1.556)	(1.541)
R-squared	0.129	0.158	0.130	0.147	0.130	0.164
Observations	14,984	14,467	14,984	14,467	14,984	14,467

***p<0.01, **p<0.05, *p<0.1; Robust standard errors in parentheses.

### 4.5 Further analysis: moderation effect testing

To thoroughly assess the impact of Adverse Childhood Experiences (ACEs) on depression among older adults, this study investigates whether pension income and the number of children during old age can lessen these effects. We included interaction terms between ACEs and both pension income and the number of children in our analytical models. As shown in **Table 6**, Model 1 indicates that pension does not significantly reduce the worsening of depression caused by ACEs in older adults. However, the results suggest that individuals with pensions have better mental health outcomes compared to those without pensions. Models 2 and 3 in **Table 6** further confirm that while receiving a pension is linked to fewer depressive symptoms, it does not offset the negative impact of childhood hardships such as parental absence or hunger on depression in late life.

**Table 6:** Testing the moderating effect of pensions.

Model	(1)	(2)	(3)
	Depression		
Medical deficiency	2.466***		
	(0.199)		
Parental absence		1.939***	
		(0.248)	
Hunger			2.854***
			(0.171)
Pension	-0.362	-0.371*	-0.480**
	(0.221)	(0.221)	(0.221)
Medical deficiency×Pension	1.377***		
	(0.338)		
Parental absence×Pension		1.714***	
		(0.338)	
Hunger×Pension			1.456***
			(0.337)
Age	0.136***	0.139***	0.130***
	(0.012)	(0.012)	(0.012)
Gender	-0.708***	-0.709***	-0.747***
	(0.158)	(0.158)	(0.157)
Marriage	-1.741***	-1.741***	-1.741***
	(0.186)	(0.186)	(0.185)
Chronic diseases	0.075	0.079	-0.104
	(0.213)	(0.213)	(0.212)
Internetuse	0.896***	0.959***	0.913***
	(0.171)	(0.171)	(0.170)
Significant accidents	-3.861***	-4.052***	-3.897***
	(0.264)	(0.264)	(0.264)
Type of housing	3.315***	3.357***	3.141***
	(0.211)	(0.211)	(0.211)
Number of permanent household residents	-1.308***	-1.404***	-1.280***
	(0.186)	(0.186)	(0.186)
Free medical check-up	-0.275***	-0.251***	-0.303***
	(0.062)	(0.062)	(0.062)
Community facilities	-0.281	-0.195	-0.393**
	(0.186)	(0.187)	(0.186)
Year	YES	YES	YES
Region	YES	YES	YES
Constant	21.046***	22.185***	21.766***
	(1.107)	(1.109)	(1.102)
R-squared	0.142	0.139	0.145
Observations	29,451	29,451	29,451

***p<0.01, **p<0.05, *p<0.1; Robust standard errors in parentheses.

**Table 7** shows the regression results with the number of children as a moderating factor. The outcomes from Models (1) to (3) in **Table 7** indicate that older adults who receive more support from their children have lower depression scores. Additionally, the amount of child support significantly lessens the negative impact of Adverse Childhood Experiences (ACEs) on elder depression, effectively reducing the harmful effect of ACEs on depressive symptoms among seniors.

**Table 7:** Testing the moderating effect of offspring support.

Model	(1)	(2)	(3)
	Depression		
Medical deficiency	2.466***		
	(0.199)		
Parental absence		1.939***	
		(0.248)	
Hunger			2.854***
			(0.171)
Pension	-0.362	-0.371*	-0.480**
	(0.221)	(0.221)	(0.221)
Medical deficiency×Pension	1.377***		
	(0.338)		
Parental absence×Pension		1.714***	
		(0.338)	
Hunger×Pension			1.456***
			(0.337)
Age	0.146***	0.148***	0.130***
	(0.012)	(0.012)	(0.012)
Gender	-0.763***	-0.760***	-0.747***
	(0.158)	(0.158)	(0.157)
Marriage	-1.740***	-1.743***	-1.741***
	(0.186)	(0.186)	(0.185)
Chronic diseases	0.295	0.293	-0.104
	(0.188)	(0.188)	(0.212)
Internetuse	0.883***	0.945***	0.913***
	(0.171)	(0.171)	(0.170)
Significant accidents	-3.942***	-4.143***	-3.897***
	(0.262)	(0.263)	(0.264)
Type of housing	3.290***	3.335***	3.141***
	(0.211)	(0.210)	(0.211)
Number of permanent household residents	-1.351***	-1.446***	-1.280***
	(0.185)	(0.185)	(0.186)
Free medical check-up	-0.268***	-0.243***	-0.303***
	(0.062)	(0.062)	(0.062)
Community facilities	-0.290	-0.201	-0.393**
	(0.186)	(0.187)	(0.186)
Year	YES	YES	YES
Region	YES	YES	YES
Constant	21.046***	22.185***	21.766***
	(1.107)	(1.109)	(1.102)
R-squared	0.142	0.139	0.145
Observations	29,451	29,451	29,451

***p<0.01, **p<0.05, *p<0.1; Robust standard errors in parentheses.

In summary, this study confirms that child support significantly lessens the negative effect of ACEs on elder depression. Conversely, no buffering role of pension benefits was found; instead, a reinforcing effect was observed.

## 5 Discussion

The negative impact of Adverse Childhood Experiences (ACEs) on the mental health of older adults appears in their increased vulnerability to depression. Life course theory suggests that childhood socioeconomic status affects adult health through its influence on economic conditions later in life, meaning that an individual’s health at any point is shaped by both current and past health experiences, which are, in turn, impacted by earlier events. Therefore, the effects of childhood socioeconomic circumstances can carry over into adulthood and old age. In line with cumulative disadvantage theory, the health outcomes seen in later life are the result of the accumulation of early life experiences (59, 63). Thus, early-life ACEs play a role in the development of depression in older age, highlighting a process that is both dynamic and accumulative. This study uses a Chinese sample to further support these theories.

Explicitly recognizing the impact of Adverse Childhood Experiences (ACEs) on depression in older adults improves our understanding of mental health issues in this group, allowing for more effective support and intervention strategies. Additionally, this paper highlights how ACEs are lasting, emphasizing the importance of protecting children from hardships like poverty and hunger throughout their lives to prevent psychological disorders. Protecting children’s early experiences requires collective efforts from governments, society, and individuals to create a safe and nurturing environment for their growth.

Heterogeneity regression results show that the effects of different ACEs on depression vary between urban and rural elderly populations. Early experiences of medical resource scarcity and hunger have a stronger impact on the depressive conditions of urban seniors, likely due to the living environment and social structure of cities. Urban areas are known for fast-paced lifestyles and intense competition, where seniors often face greater work and life pressures during their youth (64). A lack of sufficient medical resources in early years can lead to undiagnosed and untreated health issues, resulting in long-term physical and mental health problems. Additionally, hunger experiences may cause malnutrition, which affects brain development and function, thereby increasing depression risk. In urban settings, this impact may be heightened by the elderly’s increased awareness of their health statuses (65). Conversely, parental absence has a more significant effect on depression among rural seniors, closely linked to the family structure and cultural background of rural areas. In these regions, families are typically the main source of emotional support for the elderly. Parental absence can lead to a lack of necessary care and guidance during upbringing, resulting in incomplete psychological development (66). Furthermore, social support systems in rural areas are relatively weaker, making it harder for seniors to receive timely assistance and support when facing difficulties (67). Therefore, parental absence has a more substantial impact on depression among rural seniors.

The regression results for the moderating effect show that elderly individuals with pensions have better depressive conditions; however, pensions do not lessen the impact of ACEs on their depression. This Chinese evidence suggests that income status in old age does not lessen the negative effects of ACEs on elderly mental health. One reason might be that early-life ACEs influence the buildup of social resources like education, which ultimately impacts income levels and other social resources later in life, negatively affecting psychological health and increasing depression scores. Additionally, this study reveals that offspring support can counteract the negative influence of ACEs on elderly depression, effectively reducing their adverse effects. This aligns with the traditional Chinese saying “The more sons and daughters, the more happiness,” indicating that having more children enhances the elderly’s sense of well-being (68). Currently, Chinese seniors mainly live in nuclear families, and the traditional belief that raising children for old age is important remains strong. Support from children can offset the negative effects of ACEs on depression. First, emotional support from children provides comfort and builds a strong family bond. This emotional connection not only strengthens family unity but also acts as an unseen shield against external stressors and loneliness. Second, financial support from children eases money worries that could cause anxiety and worsen depressive symptoms. With financial security, the elderly can face life’s difficulties more calmly, preventing their depression from worsening. Third, daily care and attention from children are crucial forms of support. Whether assisting with daily tasks or checking on health issues, children’s support helps elderly individuals feel cared for and safe. This not only improves their quality of life but also boosts their resilience, giving them more courage and strength to manage past traumas.

## 6 Conclusion

This study provides strong evidence of the lasting effects of adverse childhood experiences (ACEs) on mental health in old age, based on a large national sample of Chinese older adults. Our findings confirm that early-life hardships, such as health problems, absence of parental support, and childhood hunger, leave a lasting impact, significantly increasing the risk of depression later in life. Importantly, this analysis goes beyond simply confirming this link to highlight resilience; while pension income, an indicator of economic security, did not influence this relationship, support from adult children emerged as a powerful protective factor, substantially lessening the psychological impact of ACEs. These results not only support the core ideas of life course theory but also have important implications for policies and interventions for China’s aging population.

The nuanced differences between urban and rural areas in our findings call for targeted policy responses. The increased sensitivity of urban elders to early-life hardships, such as medical shortages and hunger, indicates that city-based policies should focus on addressing the long-term health effects of these issues. This can be done through community programs that provide better nutritional support and comprehensive health screenings for older adults who experienced adversity early in life, ensuring early detection and management of chronic conditions linked to childhood hardships (69). Conversely, the significant impact of parental absence on rural elders highlights a deep emotional wound, likely caused by the historical context of labor migration and family separation. For rural areas, policies should prioritize improving mental health services and building community support networks that offer emotional care and help compensate for weakened family ties. Such geographical tailoring is crucial for effectively addressing the different legacies of childhood adversity across China’s varied socioeconomic landscape.

The strong moderating effect of offspring support emphasizes the essential role of the family unit within Chinese culture and aligns with research on the protective nature of social bonds. This indicates that policies designed to improve mental health among older adults should actively support, rather than inadvertently weaken, family caregiving. Governments might consider financial incentives, such as tax credits or direct subsidies, for families caring for elderly parents, thereby recognizing and bolstering the importance of filial piety (70). Additionally, workplace policies that offer flexible work arrangements and eldercare leave can help adult children better fulfill their supportive roles without facing significant financial hardship. Nonetheless, it is also important to acknowledge the potential stresses on caregivers; therefore, policies should also promote the growth of a professional community care sector to serve as a complementary support system, providing respite care and skilled services that reduce family burdens and foster sustainable care models (71).

Despite these implications, interpretations of our findings should be tempered by awareness of the study’s limitations. Mainly, the observational nature of our data, even with propensity score matching to control for observable confounders, prevents us from making definitive causal claims. The associations we find, though strong, may be affected by unmeasured confounding variables. As is typical in retrospective life-course research, measuring adverse childhood experiences (ACEs) is vulnerable to recall bias, where individuals might inaccurately remember past events (72). Additionally, our way of defining ACEs, while capturing key material and relational deprivations, is not exhaustive and excludes other forms of adversity like abuse or neglect. Future longitudinal studies, tracking cohorts from childhood into old age, are needed to establish causality clearly and to examine biological mechanisms such as allostatic load or epigenetic changes that link early stress to health outcomes later in life. Exploring a wider range of adversities and resilience factors, including social participation and psychological resilience, will deepen our understanding of this complex process.

In conclusion, this study explains how childhood adversity can negatively impact later years, but more importantly, it shows how strong family bonds can foster resilience. The study recommends a policy approach with two main focuses: one that addresses the different effects of early-life trauma across various regions, and another that boosts families’ ability to provide support while also improving the professional care system. By adopting such a comprehensive, life-course strategy, China can better protect the mental health of its rapidly aging population, ensuring that a tough start in life doesn’t determine a less satisfying old age.

## Data Availability

The original contributions presented in the study are included in the article/supplementary material. Further inquiries can be directed to the corresponding author.

## Appendix A. Description of the 11-item CES-D Scale Used in the Study

The following 11 questions were used to measure depressive symptoms. Respondents were asked: “How often have you felt this way during the past week?”

1. Have you experienced a positive mood? (Positive item)

2. Have you felt sadness?

3. Have you perceived your days as good? (Positive item)

4. Have you experienced a lack of appetite?

5. Have you had difficulty sleeping?

6. Have you felt a sense of worthlessness?

7. Have you felt a lack of purpose or things to do?

8. Have you found life to be enjoyable or interesting? (Positive item)

9. Have you felt a lack of companionship?

10. Have you felt ignored by others?

11. Have you felt socially isolated?

Response Options and Scoring:

No (0 points)

Sometimes (1 point)

Often (2 points)

Positive items (1, 3, 8) were reverse-scored (i.e., No=2, Sometimes=1, Often=0).The total score was calculated by summing the points for all 11 items, resultingin a potential range of 0 to 22, with higher scores indicating more severe depressive symptoms.
